# Vacuolar targeting of recombinant antibodies in *Nicotiana benthamiana*

**DOI:** 10.1111/pbi.12580

**Published:** 2016-06-14

**Authors:** Carolina Gabriela Ocampo, Jorge Fabricio Lareu, Vanesa Soledad Marin Viegas, Silvina Mangano, Andreas Loos, Herta Steinkellner, Silvana Petruccelli

**Affiliations:** ^1^ Centro de Investigación y Desarrollo en Criotecnología de Alimentos (CIDCA)Consejo Nacional de Investigaciones Científicas y Técnicas (CONICET)Departamento de Ciencias BiológicasFacultad de Ciencias ExactasUniversidad Nacional de La PlataLa PlataArgentina; ^2^ Department of Applied Genetics and Cell BiologyUniversity of Natural Resources and Life SciencesViennaAustria; ^3^ Present address: Fundación Instituto LeloirAv. Patricias Argentinas 435Buenos AiresArgentina; ^4^ Present address: Aridis Pharmaceuticals Inc.5941 Optical CourtSan JoseCA95138USA

**Keywords:** immunoglobulin, N‐glycosylation, vacuolar sorting signals, secretory pathway, vacuolar transport, molecular farming

## Abstract

Plant‐based platforms are extensively used for the expression of recombinant proteins, including monoclonal antibodies. However, to harness the approach effectively and leverage it to its full potential, a better understanding of intracellular processes that affect protein properties is required. In this work, we examined vacuolar (vac) targeting and deposition of the monoclonal antibody (Ab) 14D9 in *Nicotiana benthamiana* leaves. Two distinct vacuolar targeting signals (KISIA and NIFRGF) were C‐terminal fused to the heavy chain of 14D9 (vac‐Abs) and compared with secreted and ER‐retained variants (sec‐Ab, ER‐Ab, respectively). Accumulation of ER‐ and vac‐Abs was 10‐ to 15‐fold higher than sec‐Ab. N‐glycan profiling revealed the predominant presence of plant typical complex fucosylated and xylosylated GnGnXF structures on sec‐Ab while vac‐Abs carried mainly oligomannosidic (Man 7‐9) next to GnGnXF forms. Paucimannosidic glycans (commonly assigned as typical vacuolar) were not detected. Confocal microscopy analysis using RFP fusions showed that sec‐Ab‐RFP localized in the apoplast while vac‐Abs‐RFP were exclusively detected in the central vacuole. The data suggest that vac‐Abs reached the vacuole by two different pathways: direct transport from the ER bypassing the Golgi (Ab molecules containing Man structures) and trafficking through the Golgi (for Ab molecules containing complex N‐glycans). Importantly, vac‐Abs were correctly assembled and functionally active. Collectively, we show that the central vacuole is an appropriate compartment for the efficient production of Abs with appropriate post‐translational modifications, but also point to a reconsideration of current concepts in plant glycan processing.

## Introduction

The synthesis of pharmaceutical and industrial proteins in plants has became a reality with numerous products on the market and a variety of technologies available and facilities installed for large‐scale production (Sack *et al*., 2015). Both, stable and transient expression systems, have been successfully used to express mAbs in different plant species, organs and subcellular compartments (De Muynck *et al*., 2010). Recombinant protein accumulation levels rely on numerous factors among them subcellular localization (Egelkrout *et al*., 2012). Immunoglobulins (Igs) are generally sorted to the apoplast which is, unfortunately, often afflicted with intense proteolysis (Benchabane *et al*., 2008; Niemer *et al*., 2014). Thus, alternative strategies to optimize protein accumulation in other cellular organelles are considered. Retention of Abs in the endoplasmic reticulum (ER) frequently results in increased yields (De Muynck *et al*., 2010). Although the plant central vacuole is considered a hostile environment for foreign protein accumulation, it has been shown that some proteins, such as human alpha‐mannosidase (De Marchis *et al*., 2013), human complement factor C5a, (Nausch *et al*., 2012), collagen (Stein *et al*., 2009) and human transglutaminase 2 (Marin Viegas *et al*., 2015), accumulate in this organelle. In contrast, in carrot suspension cell cultures, secretory versions of human IgG1 and G4 have higher yields than ER and vacuolar variants (Shaaltiel *et al*., 2012). Accumulation of full‐length antibodies sorted to vacuoles in leaves has not been studied.

The plant secretory pathway is more complex than other eukaryotic organisms. Multiple distinct vacuolar compartments with either storage or degradative functions coexist in the same cell (Jauh *et al*., 1998; Paris *et al*., 1996). Small individual vacuoles fuse with each other to form larger vacuoles when different cellular processes take place (Zhang *et al*., 2014). It is well known that cargo proteins can be transported to vacuoles by different transport routes. The conventional vacuolar trafficking pathway involves ER export via coat protein complex II (COPII) vesicles and subsequent Golgi and post‐Golgi transport (Xiang *et al*., 2012). In addition, a direct transport from the ER to the vacuole that is independent of COPII vesicles has also been described (De Marchis *et al*., 2013; Viotti *et al*., 2013). Proteins destined to vacuoles have different vacuolar sorting signals (VSSs) that can be either sequence specific (SS) (NPIXL or NPIR motif) and work independent of its location on the protein sequence or hydrophobic C‐terminal signals (Ct) (Matsuoka and Neuhaus, 1999; Vitale and Raikhel, 1999). These VSSs are specifically recognized by vacuolar sorting receptors (VSRs) that are located in Golgi and post‐Golgi compartments, suggesting similar mechanisms to the clathrin‐coated vesicles (CCV) pathway found in other eukaryotic cells (Hinz *et al*., 2007; Niemes *et al*., 2010). Nevertheless, it has been reported that interaction of vacuolar cargo with VSR is initiated in the ER (Niemes *et al*., 2011), which supports the conventional model for vacuolar protein sorting (Robinson and Pimpl, 2013; Xiang *et al*., 2012).

N‐glycosylated proteins, such as immunoglobulins, are well suited molecules to study vacuolar targeting mechanisms as the N‐glycosylation status allows conclusions on intracellular trafficking processes. Protein N‐glycosylation starts in the ER with the transfer of the oligosaccharide precursor Glc_3_Man_9_GlcNAc_2_ to specific asparagine (Asn) residues of the polypeptide followed of a limited trimming in both the ER and Golgi and sequential addition of monosaccharides, as the protein travel through the Golgi complex, to yield complex N‐glycans, typically GlcNAc_2_Man_3_XylFucGlcNAc_2_ (GnGnXF) structures (Castilho and Steinkellner, 2012). Further modifications of the oligosaccharides include the addition of galactose beta‐1,3 and fucose alpha‐1,4 linked to the terminal GlcNAc forming the called Lewis A oligosaccharide structure for apoplast proteins (Fitchette‐Lainé *et al*., 1997; Strasser *et al*., 2007). In addition, paucimannosidic Man_3_XylFucGlcNAc_2_ (MMXF), which derives from the removal of terminal GlcNAc residues from complex N‐glycans, is present. The formation of such plant typical structures has long been assigned to be vacuolar specific (Gomord *et al*., 2010; Lerouge *et al*., 1998); however, recent characterization of the enzymes responsible for the cleavage of terminal GlcNAc residues (i.e. hexosaminidases) (Liebminger *et al*., 2011) and the presence of significant amounts of MMXF structures in the plant apoplastic fluid (Schneider *et al*., 2015) point to a broader distribution. Interestingly, the expression of a vacuolar sorted IgG in tobacco BY2 cells showed an increased level of MMXF structures when both heavy chain (HC) and light chain (LC) are fused to sporamin ssVSS in comparison with when only HC is fused (Misaki *et al*., 2011).

In this work, we studied the impact of vacuolar deposition of an IgG antibody in *Nicotiana benthamiana* leaves. Thus, we fused two different VSSs derived from the amaranth 11S globulin (KISIA Ct and the NIFRGF ss) to a mAb, to evaluate vacuolar accumulation as alternative production strategy. Further, we aimed to elucidate so far poorly understood mechanisms of vacuolar trafficking pathways and N‐glycan processing in this subcellular compartment.

## Results

### Transient expression of the 14D9 mAb variants in *Nicotiana benthamiana* leaves

**Figure 1:**
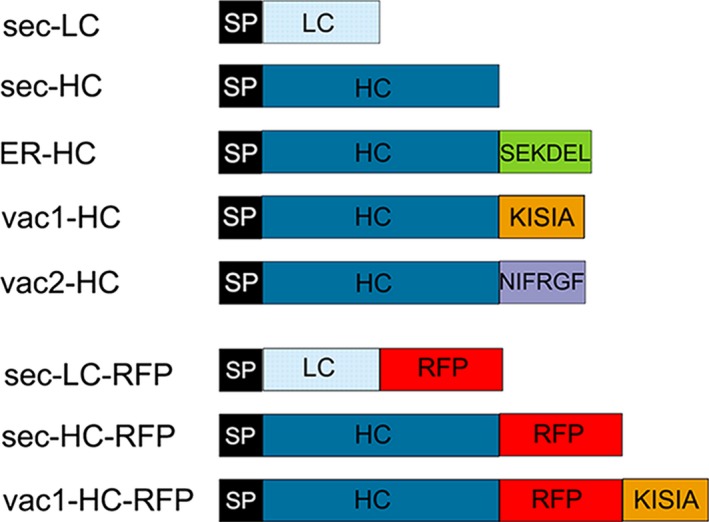
Schematic representation of the 14D9 monoclonal antibody constructs used for *Agrobacterium*‐mediated transient expression in *Nicotiana benthamiana* leaves. Proteins were introduced in the secretory pathway with gamma‐1 murine signal peptide (SP). No further sorting signal was introduced for light chain (LC) and heavy chain (HC) secretory (sec‐) versions, while SEKDEL, ER retention signal peptide; KISIA CT vacuolar targeting signal (VSS) and NIFRGF sequence‐specific (ss) VSS of the amaranth 11S globulin were fused to HC to give ER‐HC, vac1‐HC and vac2‐HC, respectively. To study sorting by CLSM microscopy, different fusions to mCherry red fluorescent protein (RFP) were also obtained. Scheme is not drawn to scale.

**Figure 2:**
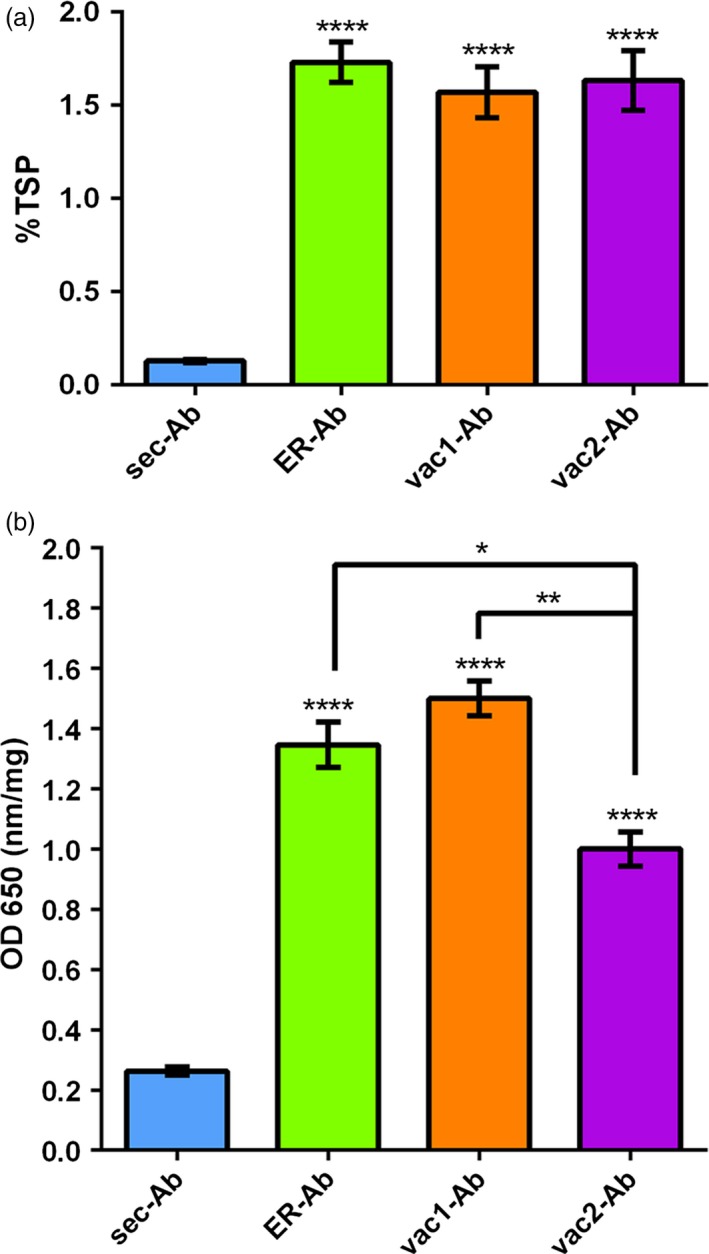
Determination of 14D9 Expression Level and Antigen Binding by ELISA. (a) Accumulation of Abs in agroinfiltrated leaves. *Nicotiana benthamiana* leaves were infiltrated with Agrobacterium carrying sec‐LC and (i) sec‐HC to produce secreted Ab (sec‐Ab), (ii) ER‐HC to generate ER‐Ab or (iii) vac1‐HC and vac2‐HC to form vac1‐Ab or vac2‐Ab, respectively. Ab amounts were quantified by ELISA of three biological replicates (each replicate containing five leaf discs of the infiltrated tissue from a different plant) and were expressed as % of total soluble protein (TSP). Error bars represent the standard error of the mean (SEM). ****Denotes statistically significant difference by Tukey comparisons test (*P* ≤ 0.0001). ER‐Ab, vac1‐Ab and vac2‐Ab protein yields were 10‐ to 15‐fold higher than sec‐Ab ones. Yields of Abs retained in the ER or sorted to vacuoles did not differ significantly. B: Recognition of BSA‐14D9 hapten. An indirect ELISA, using the same amount of soluble leaf protein per well, obtained as described above, was performed. The values were expressed as OD at 650 nm per mg of protein. Data represent the mean value (± SEM) of three independent ELISA experiments. The four Ab variants recognized the hapten indicating that the immunoglobulin fold properly. Statistically significant difference by Tukey comparisons test: ****P ≤ 0.0001; **P ≤ 0.01; *P ≤ 0.05.

To study the impact of subcellular targeting strategies on the accumulation of a full‐length IgG, the light chain (LC) carrying the native signal peptide (sec‐LC) of the monoclonal antibody 14D9 was combined with different sorted versions of the heavy chain (HC), as is shown in Figure 1. The secretory (sec‐HC) and the reticulum endoplasmic (ER‐HC) versions of the HC, generated recently, were used as references (Petruccelli *et al*., 2006); HC of 14D9 was C‐terminally fused to well‐characterized vacuolar targeting signals, that is KISIA (vac1‐HC) and NIFRGF (vac2‐HC) derived from the amaranth 11S globulin (Petruccelli *et al*., 2007) (Figure 1). Transient expression experiments in *Nicotiana benthamiana* leaves were performed by infiltration of agrobacteria carrying sec‐LC and the different HC variants: (i) sec‐HC to produce secreted Ab (sec‐Ab), (ii) ER‐HC to generate ER‐Ab and (iii) vac1‐HC and vac2‐HC to form vac1‐Ab and vac2‐Ab, respectively. Accumulation levels of assembled Abs were analysed by sandwich ELISA, using agroinfiltrated leaves from five different plants for each biological replicate and at least three independent experiments. Maximal expression levels were obtained between 5 and 8 days post infiltration (d.p.i). ELISA data exhibited a similar expression level of ER‐ and vac‐Abs (1.57%–1.73% of TSP) while sec‐Ab accumulation is 10‐ to 15‐fold lower (0.13 ± 0.02%TSP). To test whether LC and HC variants were assembled into functional antibodies, the recognition of 14D9 to the corresponding antigen (i.e. BSA hapten) was evaluated by indirect ELISA. The four Ab variants were able to recognize the hapten (Figure 2b), and the obtained signal showed a good correlation with the accumulation levels of each Ab variant (Figure 2a).

**Figure 3:**
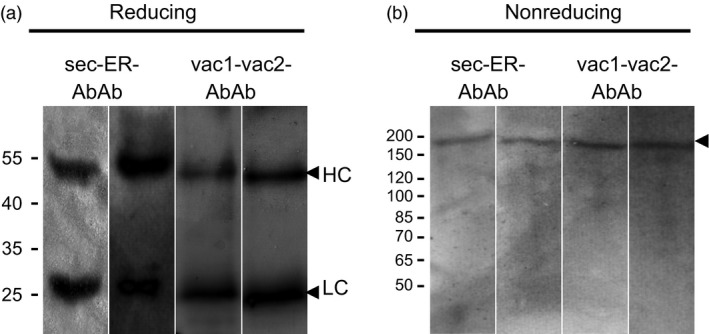
Immuno detection of purified Abs. SDS‐PAGE was performed under reducing (a) and nonreducing (b) conditions and detected by goat anti‐mouse IgG serum. Abbreviation corresponds to Figure 2a. Black arrows indicate assembled IgG (170 kDa), HC (52 kDa) and LC (25 kDa). Molecular masses are given in kilo‐dalton.

Antibodies were purified from agroinfiltrated leaves using protein G affinity chromatography and subsequently analysed by immunoblotting using anti‐mouse Ig serum for detection. Under reducing conditions, two bands of ≈ 25 and 52 kDa were detected (Figure 3a) corresponding to LC and HC, respectively. Under nonreducing conditions, the four Ab variants gave only one high‐molecular mass form at ≈ 170 kDa (Figure 3b), confirming that the four variants of the HC were able to assemble with the sec‐LC into heterotetramer and that assembled Abs can be purified from leaves.

### N‐linked glycosylation pattern of 14D9

N‐glycan profiles of purified Abs were determined by LC‐ESI‐MS as described recently (Stadlmann *et al*., 2008). The mass spectrum for the glycopeptide EEQFNSTFR (1157.52 Da) which carries the single glycosite of 14D9 (Asn297 located in the Fc domain) from the different Ab variants is shown in Table 1. The sec‐Ab exhibited predominantly complex N‐glycans composed of GnGnXF, GnF and GnGn structures (73%) typical for Golgi‐processed oligosaccharides (Table 1). Notably, 27% of oligomannosidic structures (Man 7‐9) were detect as well. In contrast, vac1‐Ab and vac2‐Ab carried mainly oligomannosidic structures (75%) typical for ER‐retained molecules (Table 1). In addition, vac‐Abs displayed about 25% GnGnXF structures. No paucimannosidic glycan structures were detected on the vac‐Ab variants.

**Table 1:** N‐linked glycans on the 14D9 variants. N‐glycosylation profile as determined by liquid chromatography–electrospray ionization–mass spectrometry (LS‐ESI‐MS). Numbers represent the presence of the different glycoforms in %

N‐glycan species	Sec‐Ab (%)	Vac‐Ab (%)
Complex (total)	73	25
GnGnXF	43	25
GnF	14	–
GnGn	16	–
Oligomann. (Man7‐9)	27	75

### Subcellular localization of the different sorted Ab variants

**Figure 4:**
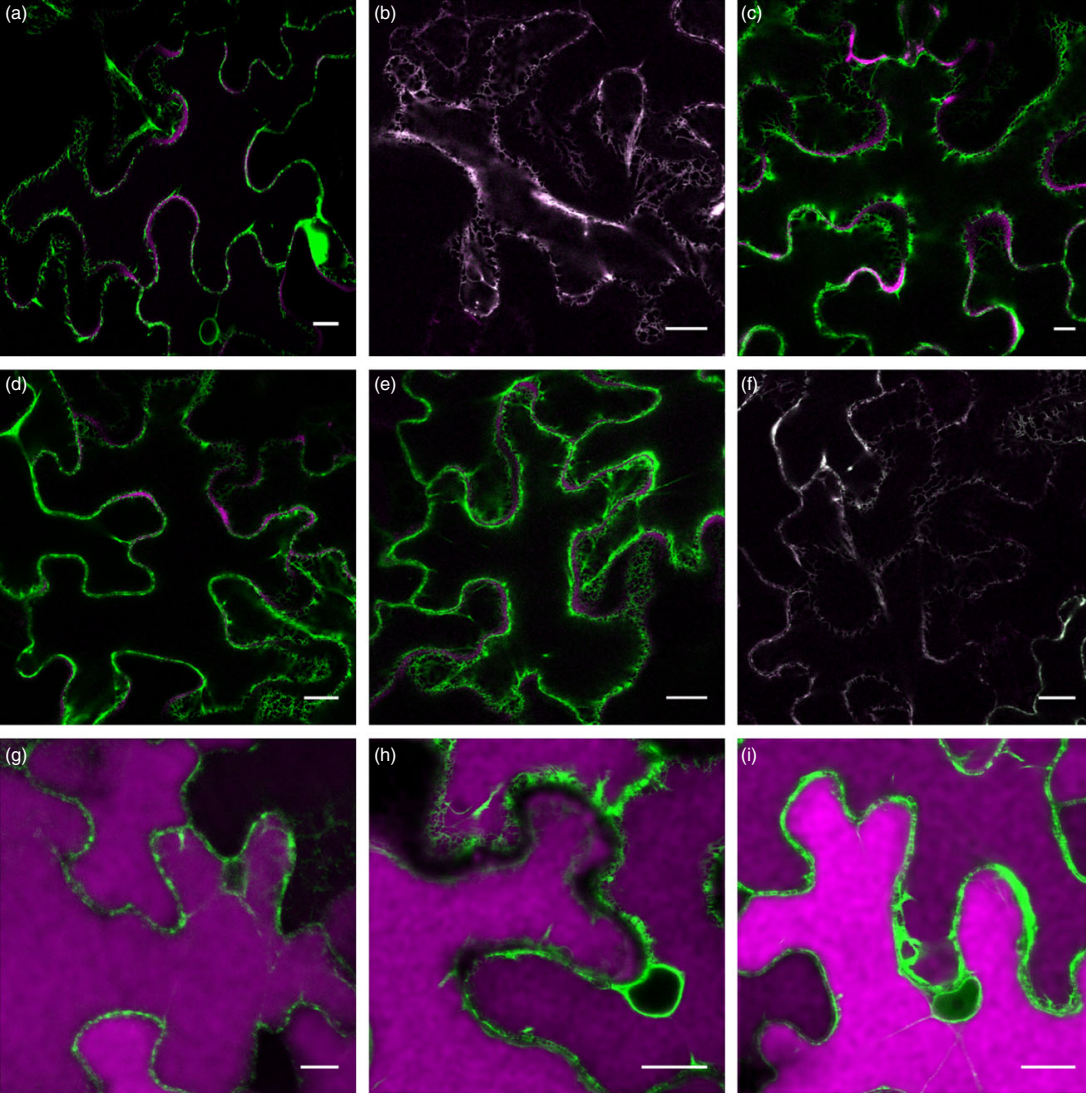
Subcellular localization of Ab variants by confocal laser scanning microscopy (CLSM). *Nicotiana benthamiana* leaves were infiltrated with Agrobacterium carrying ER‐GFP, and different combinations of HC‐ and LC‐RFP fusions (see Figure 1) are as follow: sec‐LC‐RFP (a), sec‐HC‐RFP (b), sec‐LC‐RFP + sec‐HC‐RFP (c), sec‐LC + sec‐HC‐RFP (d), sec‐LC‐RFP + sec‐HC (e), sec‐LC‐RFP + ER‐HC (f), sec‐LC‐RFP + vac1‐HC (g), sec‐LC‐RFP + vac2‐HC (h), sec‐LC‐RFP + vac1‐HC‐RFP (i). FP inspection in agroinfiltrated epidermal cells was performed 5 d.p.i. The images correspond to the merge channel resulting from the combination of RFP fusions (showed in magenta) and GFP (showed in green). Colocalization is shown in white colour. (a) sec‐LC‐RFP has an irregular red fluorescent pattern typical of apoplast localization. (b) sec‐HC‐RFP has a typical network ER pattern. (c) sec‐LC‐RFP + sec‐HC‐RFP showed an apoplast pattern. (d,e) sec‐LC + sec‐HC‐RFP and sec‐LC‐RFP + sec‐HC showed an apoplast staining pattern, respectively. (f) sec‐LC‐RFP + ER‐HC exhibited typical ER staining. (g) sec‐LC‐RFP + vac1‐HC, (h) sec‐LC‐RFP + vac2‐HC, (i) sec‐LC‐RFP + vac1‐HC‐RFP. (g–i) showed a strong red fluorescence on central vacuoles, no colocalization with ER‐GFP was detected around the nucleus. Scale bars 15 μm.

Different Ab fusions to RFP were generated (Figure 1), and protein deposition was monitored by confocal laser scanning microscopy (CLSM). To confirm that RFP fusions do not affect Ab sorting, expression of the different LC‐ and HC‐RFP fusions was analysed. Figure 4a shows that sec‐LC‐RFP has an irregular red fluorescent pattern on the border of leaf epidermal cells typical of apoplast localization (red fluorescent channel showed in magenta colour). No colocalization with ER‐GFP was observed. In contrast, sec‐HC‐RFP exhibited a typical network ER‐pattern and colocalizes with ER‐GFP (Figure 4b, white colour derives from the merge of RFP magenta and GFP green channels). This is in agreement with the proposed model for *in vivo* assembly of Ig stating that CH1 domain is unable to fold when LC is not present and therefore remains in the ER (Feige *et al*., 2009). When both sec‐LC‐RFP and sec‐HC‐RFP were coexpressed, an apoplast staining pattern was obtained (Figure 4c), confirming that the RFP fusion did not affect the final destination of sec‐Ab‐RFP. Similarly, sec‐LC and sec‐HC‐RFP (Figure 4d) or sec‐LC‐RFP and sec‐HC (Figure 4e) exhibited apoplast staining. When sec‐LC‐RFP was coexpressed with ER‐HC (Figure 4f), a typical ER fluorescent pattern was obtained. Coexpression of sec‐LC‐RFP with vac1‐HC (Figure 4g), vac2‐HC (Figure 4h) and vac1‐HC‐RFP (Figure 4i) showed strong red fluorescent signals on central vacuoles, and no colocalization with ER‐GFP was detected, not even around the nuclear envelope, where particularly strong signals were obtained (nuclear envelope has only green fluorescent). To estimate the efficiency of the vacuolar sorting, a quantitative analysis was performed. Red fluorescence in central vacuole was detected in 76.6% ± 8.3 and 79.4% ± 11.5 of the epidermal cells expressing vac1‐Ab and vac2‐Ab, respectively (Figure S1), confirming that both proteins were efficiently transported to the vacuole. In addition, the presence of vacuolar red fluorescence in leaves infiltrated with agrobacteria carrying sec‐Ab and ER‐Ab constructs was analysed and in both cases it was <5%. Taking these results into account, it can be anticipated that the observed oligomannosidic structures of vac‐Abs were not due to partial retention in the ER, but rather derive from vacuolar‐located Abs.

**Figure 5:**
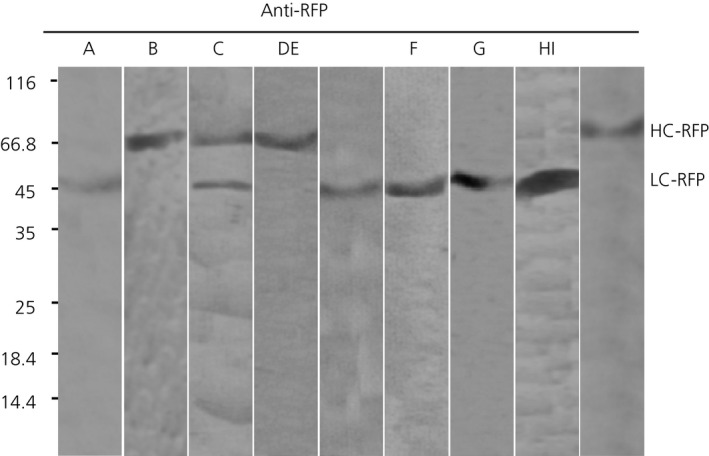
Monitoring integrity of FP fusions by immunoblot analysis. Total extracts of leaf infiltrated as described in Figure 4 were separated by SDS‐PAGE under reducing conditions, and FP fusions were monitored by anti‐RFP antibodies. Lane A: sec‐LC‐RFP, Lane B: sec‐HC‐RFP. Lane C: sec‐LC‐RFP + sec‐HC‐RFP, Lane D: sec‐LC + sec‐HC‐RFP, Lane E: sec‐LC‐RFP + sec‐HC, Lane F: sec‐LC‐RFP +ER‐HC, Lane G: sec‐LC‐RFP + vac1‐HC, Lane H: sec‐LC‐RFP + vac2‐HC, Lane I: sec‐LC‐RFP + vac1‐HC‐RFP. Molecular masses are given in kilodalton.

To verify that red fluorescence signal correspond to intact LC‐RFP and HC‐RFP fusions, an immunoblot analysis with RFP‐specific antibody was performed (Figure 5). Only bands of ~50 kDa and ~77 kDa corresponding to LC‐RFP and HC‐RFP, respectively, were detected for the different combinations of LC and HC (Figure 5), confirming the integrity of LC‐RFP and HC‐RFP fusions. In consequence, it can be anticipated that red fluorescent staining in Figure 4 corresponds to the intact Abs‐RFP fusions.

**Figure 6:**
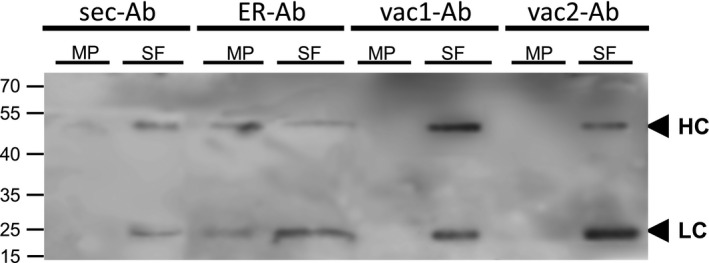
Antibody detection by microsome isolation. Leaves were infiltrated with agrobacterium carrying sec‐LC + sec‐HC (sec‐Ab), sec‐LC+ ER‐HC (ER‐Ab), sec‐LC+ vac1‐HC (vac1‐Ab) or sec‐LC + vac2‐HC (vac2‐Ab) and grounded in sucrose‐containing extraction buffer. Soluble fraction (SF) and microsomal pellet (MP) were isolated by differential centrifugation as described in Experimental Procedures. MP and SF were separated by SDS‐PAGE under reducing conditions and incubated with anti‐mouse IgG serum. Sec‐Ab, vac1‐Ab and vac2‐Ab were detected only in SF while ER‐Ab is present in both SF and MP. Black arrows indicate HC (52 kDa) and LC (25 kDa). Molecular masses are given in kilodalton.

As the strong red fluorescence in the central vacuole can overlay the ER pattern, microsomes were isolated from leaves infiltrated with the four Ab variants and the obtained fractions were analysed by immunoblot. Figure 6 shows that both vac1‐Ab and vac2‐Ab and also sec‐Ab were only detected in the soluble fraction (SF). On the other hand, ER‐Ab was found in both the microsomal pellet (MP) and the SF, similar to the results observed for other ER resident proteins such as BiP (Abas and Luschnig, 2010; Yamamoto *et al*., 2008). These biochemical data confirmed that only the ER‐Ab was located in the ER while the other Ab variants were absent in microsome fraction; therefore, oligomannosidic structures in vac‐Abs were not due to ER retention.

## Discussion

Currently, the most advanced platform for the production of IgG antibodies in plants is tobacco leaves using either transient or stable transformation systems (Ma *et al*., 2015; Mapp Biopharmaceuticals, 2015). In leaves, Abs are generally sorted to the apoplast, an environment that favours protein degradation (Benchabane *et al*., 2008; Niemer *et al*., 2014). Different strategies have been assayed to prevent apoplast degradation (Goulet *et al*., 2012; Jutras *et al*., 2015; Robert *et al*., 2013), but satisfying solutions are elusive. Thus, sorting of immunoglobulins to other subcellular compartments is a lively alternative. In this work, a depth analysis of the impact of vacuolar targeting of a full‐length Abs was performed. We showed that vacuolar variants of the mAb14D9 accumulated at levels 10‐ to 15‐folds higher than secretory forms. Although ER retention increased antibody yields in some cases (Juarez *et al*., 2013; Ko *et al*., 2005; Petruccelli *et al*., 2006; Schouten *et al*., 1996), accumulation of Ig sorted to central vacuole in leaves has not been previously reported. Only one report compared apoplast and vacuolar accumulation of mAbs in carrot cell suspension culture, and the highest yield was obtained for secreted forms of human IgG 1 and IgG 4 (Shaaltiel *et al*., 2012). Even though proteolytic enzymes have been reported to reside in the vacuoles (Jaquinod *et al*., 2007; Muntz, 2007), protein degradation dependent on various factors such as environment conditions (e.g. pH) that influence proteolytic activity. For example, vacuolar GFP is prone to proteolysis as a consequence of light‐dependent vacuole acidification but stable under dark conditions (Tamura *et al*., 2003). Here, we showed that a mouse IgG accumulated at increased yields in leaf central vacuole indicating low‐Ab‐related proteolytic activities in this compartment. Similar results were reported for human α‐mannosidase (De Marchis *et al*., 2013), human complement factor C5a, (Nausch *et al*., 2012), collagen (Stein *et al*., 2009), human α1‐proteinase inhibitor (Jha *et al*., 2012) and human transglutaminase 2 (Marin Viegas *et al*., 2015).

Notably, we demonstrate the robustness of FP fusions for the subcellular visualization of Abs, including the central vacuole. Notwithstanding, such fusions have to be performed with great care as they might interfere with Ab properties. In mammalian cells, fusion of IgG to citrine (yellow fluorescent protein) but not GFP was produced without interfering in folding, secretion and functionality of the IgG (Haas *et al*., 2010). In plants, little is known about the impact of FP fusions. Fusions of monomeric RFP and yellow Venus fluorescent protein to mAb 2G12 were not able to reach the apoplast but being retained in prevacuolar compartments (Irons *et al*., 2008). At difference of such observations, we showed that the mAb14D9‐RFP fusions sorted to the assigned final destinations.

The predominant presence of complex N‐glycan structures (GnGnXF, GnGn and GnGnF) on sec‐Ab indicates its transport through the Golgi apparatus. Our CLSM studies demonstrated that different versions of secreted Ab‐RFP fusions reached the apoplast. No colocalization of sec‐Ab‐RFP with ER‐GFP was detected, indicating that only traces if at all of Abs were in transit or ER retained. In addition, immunoblot analysis of the microsome fraction also indicates that sec‐Ab was not located in the ER. Thus, detected oligomannosidic fractions (approx. 27%) might be due to a limited exposure of the Fc glycosylation site to further glycan‐modifying enzymes. Similar results have been obtained for other secretory antibodies produced in plants (Loos *et al*., 2011a,b; Westerhof *et al*., 2014). Interestingly, vac‐Abs carried two types of glycans, predominantly oligomannosidic structures (75%) and a significant portion of complex glycans (25%). As vac‐Abs were detected exclusively in central vacuoles by CLSM and immunoblot analysis of the microsome fraction, with no signs of ER retention, it can be anticipated that these Abs variants reached the vacuole following two different trafficking routes: a direct transport from the ER to the vacuole (these Abs carry Man structures) and a Golgi‐mediated transport for Ab molecules decorated with GnGnXF. These data point that although vacuolar targeting increase protein yields, only 25% vac‐Ab molecules displayed fully processed N‐glycans, a formation often required for efficient therapeutic applications.

Immunoblot analysis and antigen‐binding studies demonstrated that vac‐Abs were correctly assembled and functionally active. It is known that unfolded or misfolded proteins in the ER can activate ER‐associated degradation (ERAD)‐releasing proteins from the ER for proteasome degradation. Also autophagy that delivers unfolded or misfolded molecules to the vacuole for degradation has been reported (Yang *et al*., 2016). Taking into account that HC and LC are assembled into functional immunoglobulin in the ER (Feige *et al*., 2009), it can be anticipated that vac‐Abs were transported to vacuoles as fully assembled molecules. Given that sec‐Abs were found in the apoplast, it is very unlikely that the transport of vac‐Abs to vacuoles is mediated by hidden VSSs in the protein sequence, as reported for a hybrid IgA/G HC (Hadlington *et al*., 2003). C‐terminal VSSs are typical of seed storage proteins that are packed into dense vesicles that drop out of the cis‐Golgi, while sequence‐specific VSSs are characteristic of lytic enzymes found in post‐Golgi compartments (Vitale and Hinz, 2005). Previous studies using nonglycosylated fluorescent proteins fused to VSS derived from storage or lytic proteins showed that these reporter proteins localized in the same prevacuolar compartments and interact to vacuolar sorting receptors in similar ways, supporting that both types of VSS sort cargo to vacuoles through the same transport pathway (Miao *et al*., 2008; Niemes *et al*., 2011). Here, using Abs as reporters that allow us to track the transit route followed via N‐glycan modifications, our results suggest that cargos having Ct‐VSS or ssVSS followed the same pathway although several trafficking mechanisms were initially inferred (Vitale and Hinz, 2005).

A direct transport route was described in pumpkin seeds where precursor‐accumulating (PAC) vesicles carried storage proteins from the ER to protein storage vacuoles bypassing the Golgi (Mitsuhashi *et al*., 2001), but in leaves, few data support a direct transport of soluble cargo. The recognition of vacuolar cargo's VSS by vacuolar sorting receptors in the ER (Niemes *et al*., 2011) and transport of human alpha‐mannosidase in tobacco leaves (De Marchis *et al*., 2013) are other proofs of the ‘nonclassical model’ proposed recently (Robinson and Pimpl, 2013). Here, we demonstrated that 75% of immunoglobulin molecules followed this direct trafficking route. Why the same IgG protein is transported by two different pathways to the vacuole in leaves is unknown, but saturation of the transport machine as occurs when IgG are overexpressed in Chinese hamster ovary (Hasegawa *et al*., 2011) might trigger the direct transport to vacuoles in plants.

The N‐glycosylation pattern of a reduced number of vacuolar glycoproteins, most of them from seeds, has been determined, and based on these studies, it was postulated that in the vacuole, the terminal GlcNAc residues from GnGn oligosaccharides are removed to produce paucimannosidic structures (Gomord *et al*., 2010; Lerouge *et al*., 1998). In addition, in carrot cells, glucocerebrosidase fused to Ct‐VSS from tobacco chitinase A has mainly paucimannosidic structures (Shaaltiel *et al*., 2007), supporting trimming of GlcNAc residues in vacuoles. Also, mouse IgG fused to the sporamin NPIRL ssVSS expressed in tobacco BY2 cells had mainly MMXF oligosaccharide (Misaki *et al*., 2011). At differences of these reports, we did not detect paucimannosidic structures in vac‐Abs produced in tobacco leaves. Similar to our findings, the secretory human IgA produced in *Nicotiana benthamiana* accumulated in intracellular compartments (protein bodies and vacuoles) but not in the apoplast and had predominantly high mannose but limited evidence for paucimannosidic structures (Paul *et al*., 2014). On the other side, significant portions of paucimmanosidic structures are detected in the apoplastic fluid representing the secretome of a plant cell (Schneider *et al*., 2015) and recombinant proteins located in the apoplast (Castilho *et al*., 2014; Dirnberger *et al*., 2001). Collectively, these data suggest a reconsideration of referring paucimannosidic structures as vacuolar specific/typical.

In conclusion, we showed that antibody deposition inside the cell, either the ER or in the vacuoles, leads to increased yields compared to apoplast accumulation. Importantly, antibodies produced in different subcellular compartments are functionally active. Although N‐glycosylation of antibodies intracellularly deposited may not suit therapeutic applications where complex structures are favoured, they might be useful for many research and biomedical applications as well as for productions of mAbs used in the downstream processing of many biologics. Other putative applications could be on synthesis of therapeutic aglycosylated antibodies with novel effectors functions (Hristodorov *et al*., 2013).

## Experimental procedures

### Plants growth and maintenance

*Nicotiana benthamiana* plants were grown for 6–8 weeks in a growth chamber at 22 °C with a 16‐h‐ligth/8‐h‐dark cycle and used for transient expression. Infiltration was performed in the third and the fourth leaves counting top‐down starting with the youngest mature leaf.

### Construction of plant antibody expression binary vectors

Plasmids containing 14D9LC (GenBank Accession Number KU933514) and 14D9HC (KU933515) are described in Petruccelli *et al*. (2006). To obtain vac1‐HC and vac2‐HC, 14D9HC was amplified by polymerase chain reaction (PCR) using oligonucleotides F‐SP (CACCATGGGCTGGAGCTGGATC) and R‐HC‐KISIA (AGCATCTAGATCAAGCAATAGAAATCTTCTCAGAACCAGGAGAGTGGGAGAG) and R‐HC‐NIFRGF (AGCATCTAGATCAGAACCCACGGAAAATGTTCTCAGAACCAGGAGAGTGGGAGAG); the amplified fragment was directional cloned into pENTR‐D‐TOPO (Life Technologies SA, CABA, Argentina) to give pENTR‐vac1‐HC and pENTR‐vac2‐HC. Then, vac1‐HC and vac2‐HC were transferred to pGWB2 destination vector (Nakagawa *et al*., 2007) using LR clonase (Life Technologies SA) to obtain pGWB‐vac1‐HC and pGWB‐vac2‐HC.

To fuse 14D9 antibody genes to fluorescent protein (FP) gene, an overlap extension polymerase chain reaction (OE‐PCR) strategy was used. To this end, LC gene was amplified with F‐SP and R‐LC‐fusFP (CTCCTCGCCCTTGCTCACCATCTCAGAACACTCATTCCTCTTGAAGCT) oligonucleotide primers and HC with F‐SP and R‐HC‐fusFP (GATGACGTCCTCGGAGGAGGCCATGGCGGCCCCTTTACCAGGAGAGTGGGAG) primers to produce LC‐fus1 and HC‐fus1 PCR products, respectively. The gene encoding the RFP Cherry from ER Cherry (CD3‐959, ABRC) (Nelson *et al*., 2007) was amplified with F‐LC‐fus‐RFP (GGTGGGTACCGGCTAGCACCAATGGTGAGCAAGGGC) and R‐FP (TTACTTGTACAGCTCGTCC) or F‐HC‐fus‐RFP (CTCCCACTCTCCTGGTAAAGGGGCCGCCATGGCCTCCTCCGAGGACGTCATC) and R‐FP using to obtain LC‐fus2 and HCfus2 PCR products, respectively. Finally, the external F‐SP and R‐FP oligonucleotide primers were used to amplify a mix of the PCR products: LC‐fus1 and LC‐fus2 or HC‐fus1 and HC‐fus 2 to produce LC‐RFP and HC‐RFP, respectively. Then, LC‐RFP and HC‐RFP PCR products were directionally cloned into pENTR‐D‐TOPO (Life Technologies SA). After that, LC‐RFP or HC‐RFP fragments, flanked by attL sites, were released from pENTR‐HC‐RFP or pENTR‐LC‐RFP by digestion *Mlu* I restriction enzyme (Promega, Fitchburg, WI), and these DNA fragments were used in the LR recombination reaction with pGW2 as destination vector (Nakagawa *et al*., 2007) LR clonases (Life Technologies SA) to obtain pGWB2‐LC‐RFP and pGWB2‐HC‐RFP.

### Agrobacterium‐mediated transient protein expression

*Agrobacterium tumefaciens* stain GV3101 cultures carrying LC, HC; LC‐FP and HC‐FP constructs were grown in YEB media (5 g/L beef extract, 1 g/L yeast extract, 5 g/L peptone, 5 g/L sucrose, 2 mm MgSO_4_) at 28 °C overnight. Cells were collected by centrifugation at 5000 ***g*** and resuspended in infiltration media, IM (100 mm MgCl_2_, 10 mm 2‐(N‐morpholino) ethanesulfonic acid (MES), pH 5.7, 200 μm acetosyringone) adjusting agrobacterium OD_600_ to 0.6 for HC and LC constructs and to 0.1 for P19 silencing suppressor of tomato bushy stunt virus (Baulcombe *et al*., 2002), followed with incubation at 28 °C for at less 3 h. The abaxial face of *Nicotiana benthamiana* leaves was pressure infiltrated with the agrobacterium suspension using a 1‐mL syringe, and then, the plants were incubated 20 °C with 16 h light and 8 h dark. For CLSM, agrobacterium OD_600_ was adjusted to 0.1 for LC‐RFP or sec‐LC, to 0.3 for HC‐RFP, sec‐HC, ER‐HC, vac1‐HC or vac2‐HC, to 0.1 for GFP‐HDEL (Brandizzi *et al*., 2003) and to 0.1 for P19 (Baulcombe *et al*., 2002). All infiltrations were performed with ER‐GFP, P19 and different combinations of LC and HC: (a) sec‐LC‐RFP, (b) sec‐HC‐RFP, (c) sec‐LC‐RFP + sec‐HC‐RFP, (d) sec‐LC + sec‐HC‐RFP, (e) sec‐LC‐RFP + sec‐HC, (f) sec‐LC‐RFP + ER‐HC, (g) sec‐LC‐RFP + vac1‐HC, (h) sec‐LC‐RFP + vac2‐HC, (i) sec‐LC‐RFP + vac1‐HC‐RFP. As control, infiltrations with agrobacteria carrying only ER‐GFP, P19 or empty pGWB2 vectors were also performed.

### Enzyme‐linked immunosorbent assay (ELISA)

To quantitatively evaluate the expression levels of the Ab variants, different sets of *N. benthamiana* plants were used for each transient expression independent experiment (three biological replicates). For each experiment, at least five plants per construct were used. Leaves were numbered from the top‐down starting with the youngest mature leaf (no.1), and leaves no. 3, 4 and 5 were infiltrated. Each construct was infiltrated in leaves located at different positions (leaf 3, 4 or 5). Leaf samples were collected at 5–8 d.p.i and stored at −80 °C until analysis. Each replicate contained five leaf pieces of the infiltrated tissue from different sets of plants that were grounded to a fine powder and suspended in extraction buffer (20 mm sodium phosphate, 0.5M sodium chloride, pH 7.5) for 15 min at 4 °C. After centrifugation at 10 000 ***g***, total soluble protein concentration in the supernatant was measured by Bradford assay (Bradford, 1976) using bovine serum albumin as standard. Each replicate sample was analysed by sandwich ELISA in triplicate as previously described (Petruccelli *et al*., 2006). Briefly, plastic wells (Maxisorp, Nunc, Denmark) were coated with 1 μg/mL of goat anti‐mouse antibody specific to LC in phosphate‐buffered saline (PBS) at 4 °C overnight. Then nonspecific binding sites were subsequently blocked with 3% (w/v) nonfat milk solution in PBS for 1 h at 37 °C. After three washes, plates were incubated with 100 μg soluble protein leaf extract overnight at 4 °C. The plates were washed again and then incubated with biotinylated goat anti‐mouse antibody specific to HC (1 μg/mL) in 1% (w/v) nonfat milk, overnight at 4 °C, followed by incubation with high‐sensitivity streptavidin‐HRP conjugate for 30 min at 37 °C. Plates were washed five times before incubation with tetramethylbenzidine (TMB) peroxidase substrate (Kirkegaard and Perry Laboratories, Gaithersburg, MD). The optical density was measured at 650 nm. A purified mouse immunoglobulin (Sigma‐Aldrich, St Louis, MO) was used as standard for the calibration curve.

To analyse the ability of the antibodies to interact with the antigen, an indirect ELISA was carried out. The mAb14D9 is a catalytic antibody that recognized 14D9 enol ether hapten (Reymond *et al*., 1993). Plastic wells were coated with 14D9 hapten coupled to BSA (1 μg/mL) (provided by Richard Lerner, The Scripps Research Institute, La Jolla CA) by passive adsorption for 16 h at 4 °C. The plates were then blocked with 3% nonfat milk solution for 1 h at 37 °C, and subsequently, total leaf extracts were added to the plates and incubated overnight at 4 °C. Finally, incubations with biotinylated goat anti‐mouse antibody, with streptavidin‐HRP conjugate and TMB peroxidase substrate were performed as described above.

### Total protein extraction and immunoblot analysis

*Nicotiana benthamiana* leaves were collected at 5–8 d.p.i., and total protein were extracted by grinding 16 leaf discs in 160 μL SDS‐PAGE sample buffer (72 mm Tris‐HCl, 2% SDS, 10% glycerol, 5% β‐mercaptethanol, pH 7). The extracts were then boiled for 5 min and centrifuged at 15 000 ***g*** for 10 min. After that, approximately 20 μg of total leaf extracts were separated by SDS‐PAGE followed by blotting to nitrocellulose membranes (Schleicher & Schuell Bioscience, Inc., Dassel, Germany). The membranes were stained with Ponceau S (Sigma‐Aldrich) to ensure equal protein loading prior to immunodetection. Nitrocellulose membranes were first blocked with 5% (w/v) nonfat milk in TBS for 1 h at 37 °C. The membranes were incubated with a biotinylated goat anti‐mouse antibody 1 : 3000 (# 31802; Thermo Scientific Pierce, Rockford, IL), overnight at 4 °C, and with high‐sensitivity streptavidin‐HRP conjugate (# 21130; Thermo Scientific Pierce) 1 : 20 000 for 30 min at 37  °C. Finally, chemiluminescence was generated by addition of 1.25 mm luminol (#A8511; Sigma‐Aldrich), 200 μm p‐coumaric acid (#C9008; Sigma‐Aldrich), 0.09% [v/v] H_2_O_2_, 0072% [v/v] DMSO, 100 mm Tris‐HCl pH 8.5 substrate, and luminescent signal was captured using X‐ray film (Amersham Hyperfilm ECL, GE Healthcare Life Sciences Argentina SA). For RFP detection, a rabbit anti‐RFP antibody (#R10367; Thermo Scientific Pierce) and goat anti‐rabbit IgG (H+L) secondary antibody biotin conjugate (#31820; Thermo Scientific Pierce) 1 : 2000 and 1 : 20 000, respectively, were used following the same procedure described above.

### Purification of recombinant IgG from agroinfiltrated leaves

Agroinfiltrated *Nicotiana benthamiana* leaves (30 g for sec‐Ab, 10 g for ER‐Ab and vacs‐Abs) were grounded with mortar and pestle in liquid nitrogen until a fine powder. Then, the tissue powder was extracted with extraction buffer (1.5 m NaCl, 45 mm Tris, 1 mm EDTA, 40 mm ascorbic acid, pH 7.5) for 15 min at 4 °C with agitation, using a ratio of 1 mL buffer per 1 g fresh leaf tissue. The leaf extracts were centrifuged three times at 10 000 ***g*** for 10 min at 4 °C. The supernatant was incubated with 20 μL of Protein G Sepharose (#17‐0618‐01GE Healthcare Life Science Argentina S.A.) for 1 h 30 min at 4 °C in gentle agitation. Subsequently, antibodies bound to Protein G Sepharose were retained in Micro Bio‐Spin columns (#732‐6204; Bio‐Rad, Hercules, CA), the column was washed three times with 800 μL of extraction buffer without ascorbic acid and finally elution was performed by addition of 50 μL of SDS‐PAGE sample buffer followed of heating at 95 °C for 5 min. Extracted antibodies were used in N‐glycan analysis as described below.

### N‐glycan analysis

The N‐glycosylation profiles of Abs were determined by LC‐ESI‐MS as previously described by Stadlmann and colleagues (Stadlmann *et al*., 2008). In brief, purified IgG was separated by reducing SDS‐PAGE, and bands corresponding to the HC were excised from the Coomassie‐stained gel. Upon S‐alkylation and tryptic or tryptic/GluC digestion, fragments were eluted from the gel with 50% acetonitrile and separated on a reversed‐phase column (150 × 0.32 mm BioBasic‐18; Thermo Scientific) using a gradient of 1–80% acetonitrile. This method generates one glycopeptide EEQFNSTFR [M+H]1+: 1157.52 Da. Glycopeptides were analysed with a Q‐TOF Ultima Global mass spectrometer (Waters, Milford, MA). Spectra were summed and deconvoluted for the identification of glycoforms. Glycans were annotated according to the proglycan nomenclature (www.proglycan.com).

### Isolation of microsomal membrane fraction

Microsomal isolation was carried out mostly as described in Abas and Luschnig (2010). All steps were performed on ice and all centrifugations were performed at 4 °C. Leaves (50 mg for ER‐Ab and vac‐Abs and 200 mg for sec‐Ab) were grounded with a extraction buffer EB (100 mm Tris–HCl, 25% [w/w] sucrose, 5% [v/v] glycerol, 10 mm EDTA, 10 mm EGTA, 5 mm KCl and 1 mm DTT, pH 7.5). About 1.0 μL EB/mg material, minimum volume 100 μL was used. The leaf extracts were centrifuged at 600 ***g*** for 3 min, and supernatant was kept. The pellet was re‐extracted using half of the original volume and centrifuged at 600 ***g*** for 3 min, and the supernatant was collected and added to the first one. The leaf extracts were centrifuged at 600 ***g*** for 3 min, and supernatant was kept. The pellet was re‐extracted with 1.1X EB and centrifuged at 600 ***g*** for 3 min, and the supernatant was collected and added to the first one. The combined supernatants were centrifuged at 600 ***g*** for 3 min. The resultant supernatant was kept aside as the cleared homogenate.

The samples were diluted to a final sucrose concentration of 12% w/w, divided into aliquots of 200 μL in 1.5 mL tubes and centrifuged at 21 000 ***g*** for 2 h. The supernatant was removed and stored as the soluble fraction (SF), and the microsomal pellet (MP) was washed with 150 μL of wash buffer (20 mm Tris–HCl [pH 7.5], 5 mm EDTA and 5 mm EGTA). Samples were recentrifuged at 21 000 ***g*** for 45 min, and the wash buffer was discarded. The MP was resuspended in 100 μL SDS‐PAGE sample buffer and boiled for 5 min.

### Confocal laser scanning microscope analysis and image processing

*Nicotiana benthamiana* abaxial epidermical cells were observed at 5 d.p.i with a Leica TSC SP5 Confocal Laser Scanning Microscope (CLSM) (Advanced Microscopy Facility, FCE, UNLP, Argentina), using a 63X (NA 1.4) oil immersion objective. RFP was excited with a HeNe 1.5 mW laser (intensity 54%) at 543 nm and detected in the 570–630 nm range. GFP was excited with an Argon 100 mW laser (intensity 24%) and detected in the 496–532 nm range. Simultaneous observation of RFP and GFP was performed by combining the settings described above in the sequential scanning facility of the microscope, as instructed by the manufacturer. All images shown were acquired using the same photomultiplier gain and offset settings. Postacquisition image processing was performed with ImageJ software (http://rsb.info.gov/ij/).

To study of the efficiency of vacuolar targeting, ten overview images with low magnification (30–35 cells per field) were taken per sample and the numbers of cells with vacuolar red fluorescence over the total number of transformed cells were counted. Data represent the average of three independent experiments performed with vac1‐Ab‐RFP (sec‐LC‐RFP + vac1‐HC) and vac2‐Ab‐RFP (sec‐LC‐RFP + vac2‐HC). As controls, also vacuolar red fluorescence of leaf infiltrated with sec‐Ab‐RFP (sec‐LC‐RFP + sec‐HC) and ER‐Ab‐RFP (sec‐LC‐RFP + ER‐HC) were also counted.

### Statistical analysis

All statistical analyses were carried out using Prism 6 (GraphPad Software, GraphPad Inc., La Jolla, CA). One‐way ANOVA test and Tukey's multiple comparisons test were used to determine means with statistical differences. A *P*‐value <0.05 was regarded as statistically significant.

## Funding

SP is a researcher from CONICET and Professor of the Facultad de Ciencias Exactas‐UNLP; CGO, VSMV and SM are fellows at CONICET. FJL is a member of the Support Staff Career (CPA) of CONICET. This research was supported by the Agencia Nacional de Promoción Científica y Tecnológica (ANPCyT) through the grants PICT2010‐2366 and PICT2015‐0010, by Universidad Nacional de La Plata (grant 11X/630) and by Argentine Ministry of Science, Technology & Innovative Production (MINCyT)‐Austria Federal Ministry of Science, Research and Economy BMWF AU/10/13.

## Supporting information


**Figure S1** Vacuolar sorting efficiency.
