# Supplementary material for: Vacuolar targeting of recombinant antibodies in Nicotiana benthamiana
Source: Plant Biotechnol J. 2016 Jun 14;14(12):2265–75. doi: 10.1111/pbi.12580 (PMC5103231; doi:10.1111/pbi.12580)
Supplement: Supplementary file 1 — Figure S1 Vacuolar sorting efficiency. [file PBI-14-2265-s001.pdf]

I

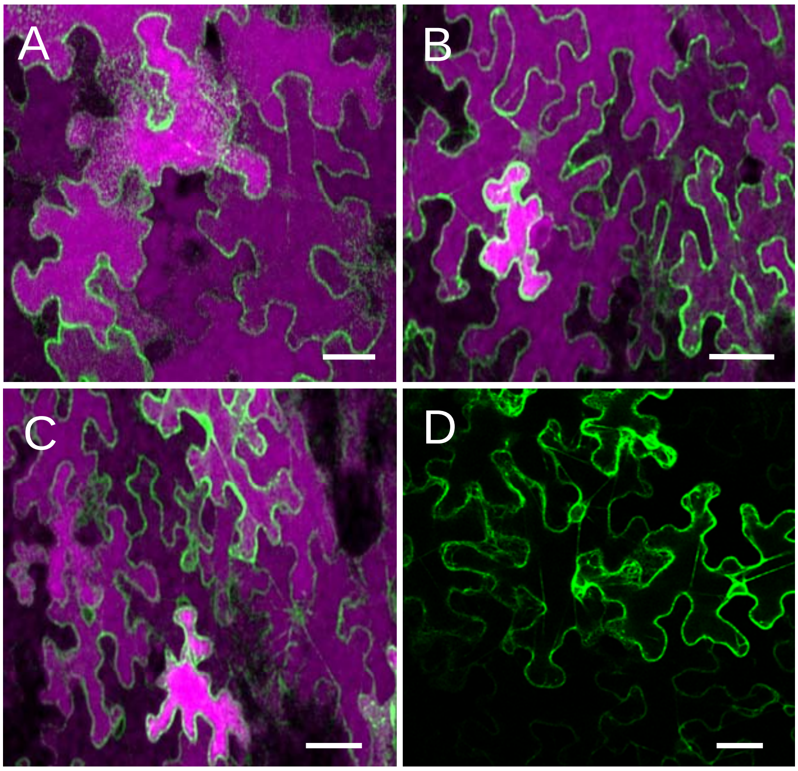

II

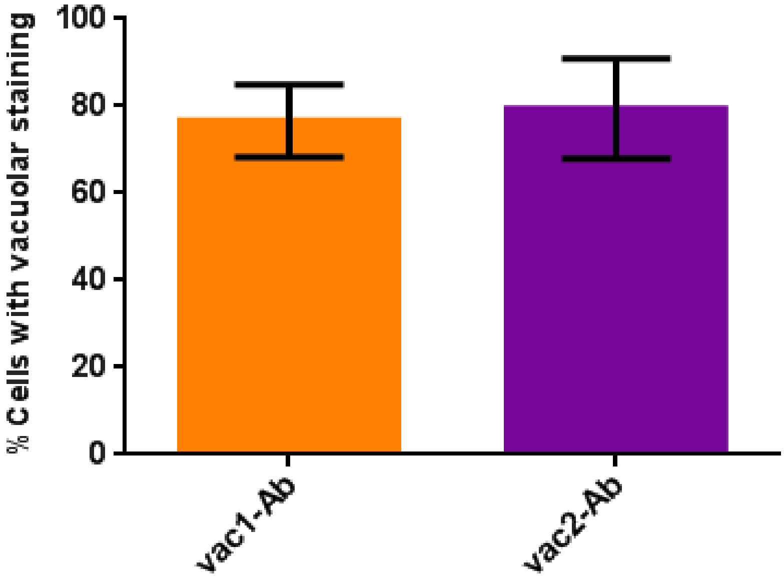

Supplementary Figure S1: vacuolar sorting efficiency

Part I: **Micrographs at low magnification of leaf epidermal cells infiltrated with agrobacterium carrying: sec-LC-RFP + vac1-HC (A); sec-LC + vac1-HC-RFP (B); sec-LC-RFP + vac2-HC (C), pGWB2 empty vector (D) obtained at 5 d.p.i. Scale bar 50 μm.**

Part II: Quantitative analysis vacuolar targeting efficiency

Ten overview image at low magnification of leaf infiltrated with sec-LC-RFP + vac1-HC or sec-LC-RFP + vac2-HC were taken and the number of cells with red fluorescence in the vacuole over the total number of cells.
